# Selective Targeting of Cancer-Associated Fibroblasts by Engineered H-Ferritin Nanocages Loaded with Navitoclax

**DOI:** 10.3390/cells10020328

**Published:** 2021-02-05

**Authors:** Leopoldo Sitia, Arianna Bonizzi, Serena Mazzucchelli, Sara Negri, Cristina Sottani, Elena Grignani, Maria Antonietta Rizzuto, Davide Prosperi, Luca Sorrentino, Carlo Morasso, Raffaele Allevi, Marta Sevieri, Filippo Silva, Marta Truffi, Fabio Corsi

**Affiliations:** 1 Dipartimento di Scienze Biomediche e Cliniche “L. Sacco”, Università di Milano, 20157 Milan, Italy; leopoldo.sitia@unimi.it (L.S.); arianna.bonizzi@unimi.it (A.B.); serena.mazzucchelli@unimi.it (S.M.); raffaele.allevi@unimi.it (R.A.); marta.sevieri@unimi.it (M.S.); filippo.silva@unimi.it (F.S.); 2 Istituti Clinici Scientifici Maugeri IRCCS, 27100 Pavia, Italy; sara.negri@icsmaugeri.it (S.N.); cristina.sottani@icsmaugeri.it (C.S.); elena.grignani@icsmaugeri.it (E.G.); carlo.morasso@icsmaugeri.it (C.M.); 3 Department of Biotechnology and Biosciences, University of Milan-Bicocca, 20126 Milan, Italy; maria.rizzuto@unimib.it (M.A.R.); davide.prosperi@unimib.it (D.P.); 4 Colorectal Surgery Unit, Fondazione IRCCS Istituto Nazionale dei Tumori di Milano, 20133 Milan, Italy; luca.sorrentino@istitutotumori.mi.it

**Keywords:** cancer-associated fibroblasts, fibroblast activation protein, targeted nanoparticles, H-ferritin, navitoclax

## Abstract

Cancer-associated fibroblasts (CAFs) are key actors in regulating cancer progression. They promote tumor growth, metastasis formation, and induce drug resistance. For these reasons, they are emerging as potential therapeutic targets. Here, with the aim of developing CAF-targeted drug delivery agents, we functionalized H-ferritin (HFn) nanocages with fibroblast activation protein (FAP) antibody fragments. Functionalized nanocages (HFn-FAP) have significantly higher binding with FAP^+^ CAFs than with FAP^−^ cancer cells. We loaded HFn-FAP with navitoclax (Nav), an experimental Bcl-2 inhibitor pro-apoptotic drug, whose clinical development is limited by its strong hydrophobicity and toxicity. We showed that Nav is efficiently loaded into HFn (HNav), maintaining its mechanism of action. Incubating Nav-loaded functionalized nanocages (HNav-FAP) with FAP^+^ cells, we found significantly higher cytotoxicity as compared to non-functionalized HNav. This was correlated with a significantly higher drug release only in FAP^+^ cells, confirming the specific targeting ability of functionalized HFn. Finally, we showed that HFn-FAP is able to reach the tumor and to target CAFs in a mouse syngeneic model of triple negative breast cancer after intravenous administration. Our data show that HNav-FAP could be a promising tool to enhance specific drug delivery into CAFs, thus opening new therapeutic possibilities focused on tumor microenvironment.

## 1. Introduction

Over the past few years, the way we approach cancer therapy has dramatically changed. Several studies have contributed to switch the focus from tumor cells to the tumor tissue as a whole, including microenvironment as an active player in determining the tumor physiology and behavior [1]. The tumor microenvironment has important biological implications on cancer initiation and progression, and it may affect tumor responsiveness to chemotherapy and control the antitumor immunity [2]. In particular, cancer-associated fibroblasts (CAFs) represent the major cell population within the tumor microenvironment, and they play a multifaceted role in various stages of oncogenesis [3,4]. Through secretion of cytokines and growth factors, CAFs mediate proliferating signals and induce drug resistance in cancer cells. Moreover, they produce an extracellular matrix that physically supports tumor cells growth, avoids penetration of drugs, and prevents access to T-cells and macrophages [5,6,7]. Considering their central role in cancer-stroma crosstalk, CAFs turned out as promising targets for novel anticancer therapeutic approaches aimed at perturbing the tumor microenvironment [8,9,10]. 

A distinctive feature of CAFs is the high expression of fibroblast activation protein (FAP), a cell surface glycoprotein of the dipeptidyl peptidase subfamily [11]. FAP expression is detected in the stroma surrounding >90% of the epithelial cancers, including malignant breast cancer, while it is not expressed in healthy tissues [12,13]. In cancer, FAP plays a role in extracellular matrix digestion and cancer invasion through its gelatinase activity; it is associated to a proangiogenic environment, and its expression has been correlated with tumor immunosuppression [14,15,16]. Clinical trials failed to provide objective benefits from specific FAP inhibition, suggesting that blocking FAP functions may be almost irrelevant for cancer as a stand-alone therapy [17,18]. However, exploiting FAP as a selective target for CAFs could still represent a promising challenge, by triggering specific and active delivery of cytotoxic drugs into these cells. Starting from this assumption, we developed a FAP-targeted nanotherapy against CAFs using a biological carrier for the pro-apoptotic drug navitoclax (Nav).

Nav (ABT-263, Abbvie) is a pro-apoptotic small molecule inhibitor of BCL-2, BCL-xL, and BCL-w [19]. It competes for the BH3-binding pocket of the pro-survival BCL-2 proteins, avoiding them to bind and sequester pro-apoptotic proteins such as BAX, BAK, PUMA, and others. In this way, Nav enables the activation of effector proteins, which induce permeabilization of the mitochondria outer membrane with subsequent induction of cellular apoptosis [20]. Phase I and II trials have been attempted with Nav as a single agent, showing limited activity in small cell lung cancer and other solid tumors [21,22]. Resistance mechanisms to Nav exist in several human cancers, including lymphoma and breast cancer [23,24,25,26,27]. As compared to highly proliferating cancer cells that frequently overexpress pro-survival factors, stromal components such as CAFs are more susceptible to cell death by Nav. Reasons for that have been attributed to the absence of MCL-1 protein and to a sort of “mitochondrial priming” in part due to upregulation of pro-apoptotic proteins [28]. This evidence has made Nav an extremely interesting compound for inducing apoptosis in myofibroblasts, also suggesting that it may function toward CAFs in the tumor microenvironment [29]. Despite being promising, the clinical use of Nav is hampered by its hydrophobic nature and by severe thrombocytopenia that makes it dose-limiting [30,31,32]. For these reasons, an agent making Nav suitable for parenteral administration and limiting its off target biodistribution might avoid these restrictions and overcome side effects. 

Here, we explored H-ferritin nanocages (HFn) as a CAF-targeted drug delivery system. HFn surface may be easily functionalized with a variety of targeting ligands, thus driving drug delivery onto desired cells with molecular precision. Moreover, the usage of HFn as a highly biocompatible protein carrier has potential for improving Nav solubility and pharmacokinetics in the bloodstream, as HFn is highly soluble and stable in biological fluids. Last but not least, HFn may help with increasing Nav intratumor accumulation due to the natural tumor homing of HFn and to the nanoparticle-mediated enhanced permeability and retention (EPR) effect [33,34]. 

The aim of the present study was to develop a FAP-targeted bionanoparticle made of HFn and loaded with Nav (HNav-FAP), and to investigate its targeting effects in cell culture in vitro. Our study revealed that the new HNav-FAP provided selective targeting of FAP-overexpressing fibroblasts over cancer cells and proved more effective in killing target fibroblasts compared to non-functionalized drugs. We also provided a preliminary proof of targeting efficiency in a syngeneic mouse model of triple negative breast cancer (TNBC) treated with functionalized HFn-FAP.

## 2. Materials and Methods

### 2.1. HFn

HFn was obtained from Molirom s.r.l. (Rome, Italy) and stored at 4 °C for the whole duration of experiments. To check stability, the protein has been periodically centrifuged (10 min, 10,000× *g*) and the concentration evaluated by absorbance analysis at 280 nm (A280, ε/1000 = 458.34, MW = 509 kDa) using a Nanodrop^TM^ 2000/2000c instrument (Thermo Fisher Scientific, Monza, Italy). 

### 2.2. HFn Loading with Navitoclax (HNav)

HNav was prepared by exploiting the metal ions affinity method, using Cu(II) as a complexing agent [35]. Nav powder (ABT-263, Purity: 99.97%, MedChemExpress, Monmouth Junction, NJ, USA; distributed by Clinisciences, Guidonia Montecelio, Italy) was dissolved in ethanol at a concentration of 4 mg/mL, aliquoted and stored at −80 °C. Nav (100 µg) was incubated in sterile conditions on an orbital shaker with 10 mM CuSO_4_ obtaining a Cu(II)–Nav complex (20 min, RT, 180 RPM). The complexed drug was added to HFn (1 mg) dissolved in a 220 mM NaCl solution and then incubated for 2 h at 180 RPM, RT. HNav was purified by gel filtration using a Zeba™ Spin Desalting Column, according to the manufacturer protocol (Thermo Fisher Scientific, Monza, Italy; Catalogue Number: 89890). The final protein concentration was assessed by Bradford assay (Thermo Fisher Scientific, Monza, Italy), while the quantity of encapsulated Nav was measured by quantitative UPLC/MS-MS analysis, as described in the following paragraph. The average number of Nav molecules per HFn nanocage has also been calculated. Results are reported as average ± SD of 27 independent experiments.

### 2.3. Determination of Nav by UPLC-MS/MS 

The quantity of encapsulated Nav was measured by UPLC/MS-MS (Waters Acquity UPLC & TQD mass spectrometer). HNav solution underwent protein precipitation by 100 times dilution in Acetonitrile, followed by 5 min vortexing and centrifugation. The supernatant was diluted 10 times with Acetonitrile-formic acid 0.2% (1/1 by volume), added with the internal standard Navitoclax^−13^C_6_ and 2 µL of the solution were injected into the UPLC/MS/MS system. The UPLC conditions were as follows: column Acquity UPLC BEH C18 1.7 μm (2.1 × 50 mm) at 30 °C; eluent A formic acid 0.2% in water, eluent B acetonitrile-formic acid 0.2%; flow rate 0.6 mL/min; linear gradient elution, 0 min 60% A until 0.4′, 2.5′ 30% A, 3′ 1% A until 4.5′, 5′ 60% A until 7′ (equilibration time). The retention time of Nav was 1.49′. The linearity of the method was assessed between 5 mg/L and 200 mg/L in HNav solution. The MS/MS conditions were as follows: electrospray interface in positive ion mode; multiple reaction monitoring acquisition, m/z 488.16 → 233.09 for quantitation, m/z 488.16 → 176.67 for identity confirmation, and m/z 491.37 →239.01 for the internal standard (CV 25, CE 12). The detection limit (signal-to-noise ratio = 3) was 0.5 mg/L. Micro-dialysis samples were processed the same way but applying an initial dilution factor of five instead of 100 for protein precipitation.

### 2.4. Preparation of Functionalized HFn-FAP and HNav-Fab

Empty and Nav-loaded HFn have been functionalized by conjugating the variable portion of an anti-Fibroblast Activation Protein antibody (Fab@FAP, Creative Biolabs Catalogue Number: TAB-024WM-F(E)) to an HFn surface. Surface conjugation of Fab fragments instead of whole anti-FAP antibodies allows to reduce the overall size of the nanoparticle and achieve selective antigen recognition. Bioconjugation was performed using a PEG-based heterobifunctional linker (Malhex-NH-PEG-O-C₃H₆-CONHS, Rapp Polymere Gmbh, Tubingen, Germany) by a two-step reaction, adapted from a previously reported procedure [36]. First, the Fab@FAP was reacted with 10-fold molar excess of the crosslinker in phosphate buffer (PBS) for 4 h RT on a rotator mixer and unreacted species were removed by washing with PBS in 30 kDa Amicon centrifugal devices (Millipore Corporate, Merck KGaA, Darmstadt, Germany). Then HFn was added to the reaction mix at 1:1 HFn:Fab@FAP molar ratio and incubated overnight (O/N) at 4 °C on a rotator mixer. To further optimize the preparation in terms of cell binding efficiency, two different lengths of the PEG linker (5 and 10 kDa) were used. The unconjugated Fab fragments were removed by washing with PBS in 100 kDa Amicon centrifugal devices (Millipore Corporate, Merck KGaA, Darmstadt, Germany) and functionalized nanoconjugates (HFn-FAP and HNav-FAP) were collected. Effective conjugation of Fab@FAP on HFn nanocage was assessed by SDS-PAGE and gel staining with the Imperial^TM^ Protein Stain (Thermo Fisher Scientific, Monza, Italy). HFn final recovery and the concentration of encapsulated Nav have been quantified by Bradford assay and by quantitative UPLC/MS-MS analysis, respectively. The average number of Nav molecules per HFn nanocage has also been calculated. Results are reported as average ± std. dev. of nine independent experiments.

### 2.5. Raman Spectroscopy

Raman spectroscopy was used to confirm actual Nav encapsulation into HFn. Raman spectra were recorded using an inVia Raman microscope from Renishaw (UK) equipped with laser light sources operating at 532/633/785 nm, as previously described [37]. First, the spectrometer was calibrated using the band of monocrystalline silicon at 520.7 cm^–1^. Raman spectra were acquired from 12 µL drops of water solution of free Nav, HFn and HNav dried on top of CaF_2_ slides (Crystran, Poole, UK) without any further preparation. Spectra were collected using a 532/633/785 nm laser line of 6.25 mW focused on the sample using a 100× objective for 10 min. The reported spectra correspond to the average of six independent acquisitions, after baseline subtraction and vector normalization.

### 2.6. Transmission Electron Microscopy

HFn, HNav and HNav-FAP were resuspended at an equivalent protein concentration of 250 µg/mL in mQ H_2_O. A 20 µL drop of suspension was spotted on a Formvar grid and let drying at RT. Then, the grid was stained with uranil-acetate 1% for 30 s at RT and dried O/N at RT [38]. Samples were evaluated by Transmission Electron Microscopy (TEM, Tecnai Spirit, FEI, Hillsboro, OR, USA) at 220–300k× magnification. 

### 2.7. Dynamic Light Scattering (DLS): Size and Zeta Potential 

DLS measurements were performed using a Zetasizer Nano Instrument (Malvern Panalytical Ltd., Malvern, UK) operating at 4 mW with a He–Ne laser (633 nm) using a scattering angle of 173°, at 25 °C, as also described in [39]. A disposable cuvette (optical path length: 1 cm) was used for the measurements of size, while folded capillary zeta cells (DTS1070, Malvern Panalytical Ltd., Malvern, UK) were used for Z-pot. The samples were dissolved in distilled water or Hepes 2 mM in order to optimize their ionic strength and be filtered before performing the analyses. Each sample was allowed to equilibrate for 30 s prior to starting the measurements.

### 2.8. Analysis of Nav Stability in Solution

Stability of Nav and HNav were evaluated by UPLC-MS/MS. Nav was first solubilized in EtOH (4 mg/mL), as done when preparing HNav. The drug was diluted either in PBS, PBS-2Captisol^®^ 20% (Ligand Pharmaceuticals Inc., San Diego, CA, USA), or EtOH at a final concentration of 100 µg/mL (1mL of final volume). The three Nav suspensions and freshly prepared HNav nanodrugs were left settling for 1 h, then 100 µL of the supernatants were pipetted into sterile tubes and analyzed as described in Section 2.3. Percentage of recovered Nav from each solution was compared to the theoretical expected concentration used for experiments, set as 100%.

### 2.9. Kinetics of Spontaneous Nav Release

Nav release from HNav and HNav-FAP was studied by microdialysis according to the manufacturer protocol (Float-A-lyzer G2 Dialysis Device, MWCO: 100 kDa, Spectrum LABS, Compton, CA, USA). To avoid any bias due to different HFn concentration, this was kept constant at 300 µg/mL in PBS. Five mL of sterile buffer were used in the collection chamber, outside the membrane. Experiments were run at 37 °C in a shaking incubator to simulate physiologic drug release. At predetermined time points (15 min, 30 min, 1 h, 2 h, 4 h, 18 h, 24 h, 48 h, 72 h, and 7 days), 5 mL of buffer were collected and replaced with fresh buffer in order to maintain sink condition. Nav concentration was measured by quantitative UPLC/MS-MS analysis from all samples (at each time point and from inside the membrane at the beginning and at the end of the analysis from the original sample). 

### 2.10. Cell Culture 

Murine cancer associated fibroblasts (CAFs) were isolated from 4T1 breast tumors generated as described below in Section 2.16. After 21 days of tumor growth, tumors were excised and dissociated using a tumor dissociation kit (Miltenyi Biotec S.r.l., Bologna, Italy; catalogue number: 130-096-730). From the single cell suspension, CAFs were isolated using the tumor-associated fibroblast isolation kit (Miltenyi Biotec S.r.l., Bologna, Italy; catalogue number: 130-116-474) according to the manufacturer protocol. Briefly, this includes a first incubation of the dissociated tumor with magnetic beads for non-tumor associated fibroblasts depletion followed by the positive selection of CD90.2-positive tumor associated fibroblasts. To check the isolation yield, the cells were fluorescently stained with CD45-FITC and CD90.2-PE antibodies and analyzed by CytoFLEX flow cytometer (Beckman Coulter, Cassina De’ Pecchi, Italy). Isolated CAFs were cultured in DMEM/Ham’s F-12 medium supplemented with 15% FBS, 2 mM L-glutamine, 100 U/mL penicillin, 0.1 mg/mL streptomycin, and 1% non-essential amino acids.

Primary human activated myofibroblasts (HMfs) were isolated from patients with Crohn’s disease undergoing ileal surgical resection, as previously described [40]. Briefly, a biopsy of strictured intestinal mucosa was digested by incubation with 1 mg/mL collagenase A (Sigma-Aldrich S.r.l., Milan, Italy), 50 ng/mL DNase I (Sigma-Aldrich S.r.l., Milan, Italy) in RPMI 1640 medium supplemented with 10% FBS, 2 mM L-glutamine, and antibiotics for 2 h at RT. After filtration through Cell Strainer 100 μm (BD Biosciences, San Jose, CA, USA), isolated myofibroblasts were seeded in tissue culture flasks and maintained in DMEM supplemented with 20% FBS, 2 mM L-glutamine, 200 U/mL penicillin, 0.2 mg/mL streptomycin, 1% non-essential amino acids, 0.25% gentamicin, and 0.4% amphotericin B. Collection of human samples was authorized by the Ethical Committee of ASST Fatebenefratelli Sacco university hospital (protocol number 0002846). All the patients signed a written informed consent prior to their inclusion in the study.

Immortalized breast cancer murine 4T1-Luc2 (Bioware Ultra, Perkin Elmer, Milan, Italy) and human MDA-MB-231 (HTB-26, ATCC-LGC Standards, Sesto San Giovanni, Italy) were cultured in RPMI 1640 and in high glucose Dulbecco’s Modified Eagle Medium (DMEM) respectively. Both media were supplemented with 10% heat inactivated fetal bovine serum (FBS), 2 mM L-glutamine, 100 U/mL penicillin, and 0.1 mg/mL streptomycin. 

All cells were grown at 37 °C in a humidified atmosphere containing 5% CO_2_ and were subcultured prior to confluence using trypsin/EDTA. Cell culture media and chemicals were purchased from Euroclone.

### 2.11. FAP and TfR1 Expression by Flow Cytometry 

FAP and TfR1 expression were evaluated by flow cytometry on 5 × 10^5^ cells/tube. For FAP, cells were pre-incubated for 15 min with blocking buffer (PBS, 2% Bovine Serum Albumin (BSA; Sigma-Aldrich S.r.l., Milan, Italy) and 2% goat serum (GS, Euroclone S.p.A., Pero, Italy)), centrifuged and then incubated with 1 µg of the Fab@FAP (Creative Biolabs, catalogue number: TAB-024WM-F(E)) for 15 min at RT. Cells were washed three times in PBS, centrifuged, and incubated with the secondary antibody (1 µg, Alexa Fluor 488 goat anti-human; Thermo Fisher Scientific; catalog number #: A-11001) in blocking buffer. For TfR1 studies, human-derived cells (MDA-MB-231 and HMfs) were incubated for 15 min with 500 µL of blocking buffer. Then, cells were centrifuged and 1 µL of anti-human TfR1 antibody (1 mg/mL, clone ICO-92, Thermo Fisher Scientific, Catalog Number #: MA1-7657) was added. Cells were washed two times with PBS, once in blocking buffer and incubated with 1 µL of secondary antibody (Alexa Fluor 488 goat anti-mouse; Thermo Fisher Scientific; catalog number #: A-11001) for 15 min at RT. Mouse-derived cells (4T1 and CAFs) were incubated with the anti-mouse CD71 antibody (Clone REA627, Miltenyi Biotec S.r.l.; catalogue number: 130-119-133), according to the manufacturer’s protocol. 

Labelled cells were washed thrice with PBS and analyzed using CytoFLEX flow cytometer (Beckman Coulter, Cassina De’ Pecchi, Italy). Acquisition was performed on 20,000 events, within the selected region of singlets viable cells. Untreated cells or immunodecorated only with the secondary antibody were used to set the region of positivity.

### 2.12. Cell Binding and Uptake Assays

To study HFn and HFn-FAP interaction with cells, the protein was labelled with fluorescein isothiocyanate Isomer I (FITC, Sigma-Aldrich S.r.l., CAS Number: 3326-32-7, Milan, Italy) according to the manufacturer’s protocol. To study how FAP functionalization regulates cell binding, cells (5 × 10^5^ cells/tube) were incubated with the different preparations at the same HFn equivalent concentration (100 µg/mL HFn in PBS-BSA 0.3%, 2 h at 4 °C). At the end of incubation, cells were washed three times in PBS and analyzed by flow cytometry, as previously described.

Intracellular uptake of optimized HFn-FAP was evaluated with HMfs. Cells were cultured until sub-confluence on glass coverslips pre-coated with collagen. Incubation was performed by adding 100 µg/mL of FITC-labelled HFn-FAP in culture medium supplemented with 1% FBS for 1 h and 3 h, at 37 °C in a humidified atmosphere containing 5% CO_2_. At the end of incubation, cells were washed three times with PBS (5 min), fixed with 4% paraformaldehyde (4%, 10 min) (Sigma-Aldrich) and permeabilized for 10 min with 0.1% Triton X-100 (Sigma-Aldrich). A blocking solution (2% BSA, 2% GS in PBS) was incubated for 1 h at RT. Then, primary antibodies were incubated with cells in the same blocking solution O/N at 4°C: mouse EEA1 (610457, BD biosciences) was used at 1:1000 dilution, while rabbit Cathepsin D (ab 75852, Abcam) was used at 1:100 dilution. After incubation cells were washed three times in PBS (5 min) and AF546 labelled secondary antibodies were incubated for 1 h (1:300, goat-anti mouse A-11003 and goat-anti rabbit A-11035 respectively, Thermo Fisher Scientific) adding 0.2 μg/mL DAPI (4′,6-diamino-2-phenylindole; Thermo Fischer Scientific) to stain cell nuclei. After three washes with PBS, coverslips were mounted in Prolong Gold antifade reagent (Invitrogen, Monza, Italy). Images were acquired with 63x magnification oil immersion lenses at 1024 × 1024 pixel resolution using the Leica SP8 confocal microscope equipped with laser excitation lines 405, 488, 546, and 633 nm.

### 2.13. Cell Extracts and Western Blotting

For the analysis of protein expression in CAFs and 4T1, cells were cultured for three passages after isolation and left to grow until sub-confluence in 6-well plates. Cells were washed twice with cold PBS, lysed in Triton lysis buffer (20 mM Tris-HCl pH 7.6, 150 mM NaCl, 1 mM EDTA, 10% Glycerol, 1% Triton X-100), containing 4% Protease Inhibitor Cocktail (Roche), 1 mM PMSF (Sigma-Aldrich), 1 mM Na_3_VO_4_ (Sigma-Aldrich), 10 mM NaF (Sigma-Aldrich), and cleared at 17,100 g for 15 min at 4 °C. 

For the evaluation of PARP-1 cleavage, cells (2 × 10^5^) cultured in a 6-wells plate were treated for 24 h with HNav or free Nav (1 μM) in culture medium. After treatment, cells were washed with PBS and lysed with 300 μL lysis buffer (2% SDS, 50 mM Tris HCl pH 7.4, 10 mM EDTA). 

Approximately 30 μg of protein from each sample were separated by SDS-PAGE and transferred onto a PVDF membrane. The membranes were blocked in TBS with 5% BSA and 0.1% Tween 20 for 1 h and then incubated O/N at 4 °C with appropriate primary antibody: rabbit polyclonal antibody to FAP (1:500, Abcam, catalog number ab28244), rabbit monoclonal antibody to α-SMA (1:1000, clone D4K9N, Cell Signaling Technology, catalog number 19245), rabbit polyclonal to Cytokeratin 19 (1:600, Abcam, catalog number ab53119), rabbit monoclonal antibody to PARP (1:1000, clone 46D11, Cell Signaling Technology, catalog number 9532), anti-α-tubulin produced in rabbit (1:1000, Sigma Aldrich, catalog number SAB2102603), and anti-GAPDH produced in rabbit (1:5000, Sigma-Aldrich, catalog number G9545). After three washes in TBS with 0.1% Tween 20, the membranes were reacted with the secondary anti-rabbit antibody conjugated with horseradish peroxidase (1:5000; Abcam) for 2 h. The bound antibody was revealed using Clarity^TM^ Western ECL Substrate (Bio-Rad), and the chemiluminescence signal was detected using the Chemidoc System (Bio-Rad). Densitometric analysis of bands was performed by ImageJ software.

### 2.14. Immunofluorescence and Confocal Laser Scanning Microscopy

Cells were cultured until sub-confluence on glass coverslips pre-coated with collagen and incubated with HNav or free Nav for 24 h at 37 °C (1 and 0.5 μM of the drug were used for the analysis of apoptotic nuclei and for staining of active BAX, respectively). After incubation, cells were washed with PBS, fixed for 10 min with 4% paraformaldehyde (Sigma-Aldrich) and then permeabilized for 10 min with 0.1% Triton X-100 (Sigma-Aldrich). A blocking step was performed for 1 h at RT with a solution containing 2% BSA, 2% GS in PBS. Activation of the pro-apoptotic protein BAX was analyzed by incubation with the anti-BAX active monomer monoclonal antibody (1 μg/mL, clone 6A7, Enzo Life Sciences, catalog number ALX-804-224) O/N in blocking buffer and Alexa Fluor 488 goat anti-mouse secondary antibody (1:300, Thermo Fisher Scientific) for 2 h at RT in blocking buffer. Nuclei were stained with 0.2 μg/mL DAPI (4′,6-diamino-2-phenylindole; Thermo Fisher Scientific). After three washes with PBS, coverslips were mounted in Prolong Gold antifade reagent (Invitrogen). Images were acquired with 40× magnification oil immersion lenses at 1024 × 1024 or 512 × 512 pixel resolution through Leica SP8 microscope confocal system equipped with laser excitation lines 405, 488, 546, and 633 nm. Apoptotic nuclei counted on DAPI-stained coverslips as a percentage of shrinked-shaped and DNA-fragmented nuclei over the total number of nuclei per field of view, as depicted in Figure S1.

### 2.15. Nav Cellular Uptake

The role of the surface functionalization in regulating drug uptake was evaluated by measuring intracellular levels of Nav in FAP-positive and FAP-negative cells. HMfs and MDA-MB-231 were seeded into 12-well plates at a concentration of 1 × 10^5^ cells/well and left adhering O/N. The following day, cells were incubated with 1 µM Nav, HNav and HNav-FAP in cell media with 1% FBS for 1 h. Cells were then washed two times in PBS and collected with Trypsin/EDTA. Cells were pelleted by centrifugation (300 g, 5 min) and lysed in 150 µL of acetonitrile and sonicating the suspension in a water bath sonicator (6 cycles of 30 min sonication + 30 min incubation on ice). The suspensions have been collected and processed for UPLC/MS-MS evaluation of Nav content. Results are reported as average ± std. dev. of three independent experiments. 

### 2.16. Cell Viability Assay

To evaluate the pharmacological activity of the nanoconstructs, cell viability was measured by MTS assay. CAFs, 4T1, HMfs, and MDA-MB-231 were treated with Nav, HNav and HNav-FAP at different concentrations (0.05, 0.25, 0.5, 1, and 2 µM of Nav in cell media with 1% FBS) for 24 h (3000 cells/well, six replicates/condition, in 96-well plates). CuSO_4_ (used to load Nav into HFn) and the empty nanocarriers (either HFn or HFn-FAP) were selected as internal controls and incubated with cells at equivalent concentrations used for Nav-loaded nanodrugs. 

At the end of incubation, cells were washed three times in PBS and incubated with 20 µL of MTS reagent (CellTiter 96^®^ AQueous One Solution Cell Proliferation Assay, Promega, Madison, WI, United States; cat number: PR-G3582) diluted into 80 µL of phenol red free DMEM (3 h, 37 °C). Absorbance was then read at 490 nm and a reference wavelength of 620 nm. Percentage of live cells was calculated with the formula (Abs _treated sample_ − Abs _blank_)/(Abs _ctrl sample_ − Abs _blank_). 

### 2.17. Animals

Animals were maintained in a fully equipped facility and used in accordance with the experimental procedures approved by the Italian Ministry of Health (aut. number 110/2018-PR). Seven-week-old female BALB/c mice were injected into the mammary fat pad with 1 × 10^5^ 4T1-Luc2 cells (Bioware Ultra, Perkin Elmer, Milan, Italy). Tumor growth was followed by caliper measurement and bioluminescence imaging [41]. 

### 2.18. Tumor Targeting and Biodistribution

After 8 days of tumor growth, mice were intravenously injected in the tail vein with 5 mg/Kg of HFn-FAP previously labeled with Alexa Fluor 660 (A20171, Thermo Fisher Scientific). In vivo imaging was performed at 1 h, 4 h, 24 h, and 48 h after IV administration of HFn-FAP using an IVIS Lumina II imaging system (PerkinElmer, Milan, Italy) with the following acquisition parameters: excitation 570 nm, 605 nm, 640 nm; emission filter Cy 5.5; exposure time 2 s; binning factor Medium; f/Stop 2; Field of View: D. Specific AF660 signal was subtracted from autofluorescence signal thanks to the spectral unmixing using the Image Math tool available with the Living Image Software 4.3.1 (Perkin Elmer, Waltham, MA, USA). The quantified epi-fluorescence signal was reported as average radiant efficiency after subtraction of the fluorescence values measured in untreated mice.

Blood was collected from the retroorbital plexus at 5 min, 15 min, 30 min, 1 h, 2 h, 4 h, 24 h, and 48 h after IV injection, using sterile glass Pasteur pipettes, collected in EDTA-treated tubes and centrifuged at 1000 g for 10 min to isolate plasma. Urine was collected into sterile tubes at 1 h, 2 h,4 h, 24 h after administration, and stored at 4°C until use. Fluorescence was evaluated using a Jasco FP8300 spectrofluorometer (Excitation 665 nm, Emission Scan 695–750 nm).

Subsequently, mice were sacrificed by cervical dislocation at 1, 4, 24, and 48 h to analyze the biodistribution and the tumor targeting of the administered HFn-FAP. The tumor and the major organs i.e., liver, kidneys, spleen, heart, lung, and brain were collected and imaged with the IVIS Lumina II imaging system as described above. Finally, all the tumors were stored at −80 °C for cryosectioning.

### 2.19. Confocal Laser Scanning Microscopy

Cryosections of 9 μm were obtained from excised tumors 1 h after HFn-FAP administration and immunostained for α-SMA. Briefly, cryosections were fixed for 5 min with 2% paraformaldehyde and permeabilized with 0.1% Triton X-100 in PBS (10 min, RT). Afterwards, samples were incubated for 1 h at RT with a blocking solution (2% BSA and 2% GS in PBS). The primary α-SMA antibody (rabbit mAb D4K9N, Cell Signaling Technology, Danvers, MA, USA) was diluter 1:100 in the same blocking solution and incubated in the dark at 4 °C O/N. After three washes in PBS, anti-rabbit Alexa Fluor 488-conjugated secondary antibodies (1:300, A32731, Thermo Fisher Scientific,) were incubated for 1 h at RT in blocking solution, adding 0.2 μg/mL DAPI (4′,6-diamino-2-phenylindole; Thermo Fischer Scientific) to stain cell nuclei. After three washes with PBS, coverslips were mounted in Prolong Gold antifade reagent (Invitrogen). Images were acquired with 20× air and 63× magnification oil immersion lenses at 1024 × 1024 pixel resolution using the Leica SP8 confocal microscope equipped with laser excitation lines 405, 488, 546, and 633 nm.

### 2.20. Statistical Analysis

Statistical analyses were conducted using two-tailed Student’s t-test in case of data that passed the Shapiro–Wilk normality test, or with the non-parametric Wilcoxon–Mann–Whitney test in case of non-normal distribution of the data. Results are expressed as means ± standard deviation (SD) or standard error (SE). The statistical significance threshold was set at *p* < 0.05.

## 3. Results

### 3.1. Cellular Model of FAP-Overexpressing CAFs 

**Figure 1:**
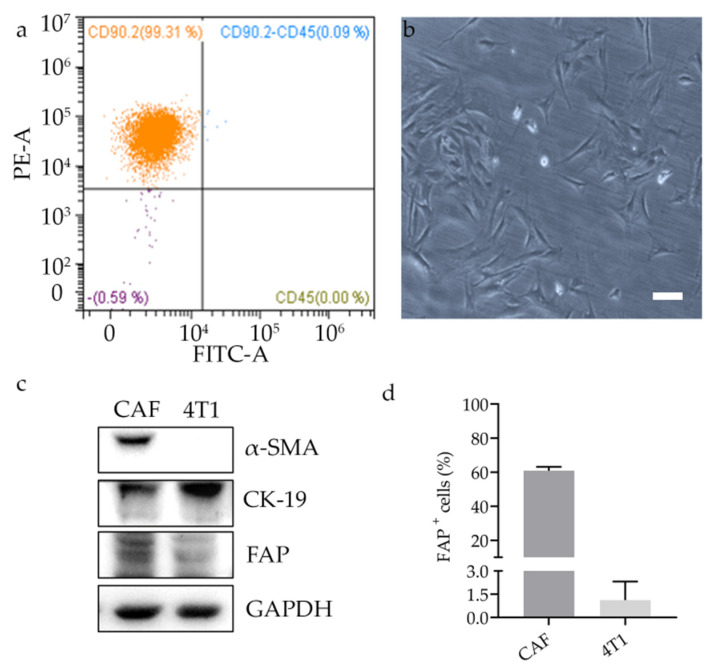
Primary culture of murine CAFs from breast cancer. Representative cytofluorimetry panel identifying the isolated CAF population in the upper left quadrant (CD45−, CD90.2+ cells) (**a**); morphology of cultured CAFs by bright field microscopy; scale bar = 20 µm (**b**); immunoblotting for FAP, α-SMA, and CK-19 in CAFs and 4T1 cells grown in culture. Loading control is represented by GAPDH (**c**); quantitative detection of FAP expression analyzed on CAFs and 4T1 by flow cytometry (**d**). Results are expressed as average percentage of positive events ± SD (n = 3).

With the aim of developing nanomedicines able to target CAFs, primary cultures of murine CAFs were isolated from dissociated 4T1 tumors grown in mice. The isolation yield and purity of obtained culture was checked by flow cytometry for enrichment in the fibroblast marker CD90.2 and disappearance of CD45 (Figure 1a, Figure S2). Due to the relative low number of CAFs (about 3%) as compared to the high number of other cells found in the tumor, we pooled three different tumors together to obtain a suitable amount of CAFs for culture establishment and expansion. Once in culture, the morphology of the cells, shown in Figure 1b, confirmed the fibroblast-like structure that can be easily distinguished from the epithelial-like structure of 4T1 cells (Figure S3a). We characterized the isolated CAFs by Western blot. As reported in Figure 1c, isolated CAFs expressed α-smooth muscle actin (α-SMA) and FAP at higher levels than 4T1 tumor cells. Both these proteins are known to be markers of CAFs. By contrast, the tumor marker cytokeratin 19 was markedly more expressed in 4T1 than in CAFs. 

To further investigate the possibility of using FAP as a selective target for CAFs, we verified its expression by cytofluorimetry both in CAFs and 4T1 cells. As it can be seen in Figure 1d, 61% of CAFs were positive to FAP staining, while less than 1% of 4T1 presented surface epitopes for this marker. This result confirmed that FAP can be a promising cell surface marker to preferentially target CAFs over tumor cells by properly designed nanodrugs. 

### 3.2. Development of Engineered HFn-FAP Nanoparticles

**Figure 2:**
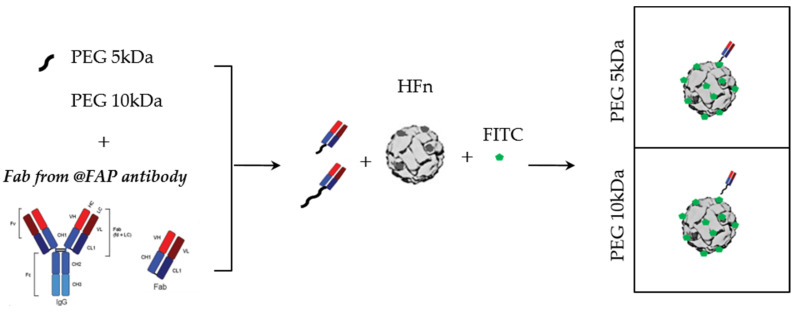
Development of functionalized HFn nanocages. Schematic representation of conjugation scheme 5 and 10 kDa) and then reacted with fluorescently labeled HFn.

HFn nanocages have been widely used by our group to deliver drugs to breast tumors [38,40]. Here, we evaluated whether we could selectively control their delivery into CAFs, by functionalizing the nanocage surface with a specific FAP targeting moiety. We functionalized HFn nanocages with the Fab fragment of an anti-FAP antibody (Fab@FAP), thus minimizing the steric hindrance on the overall nanocage size. As illustrated in Figure 2, the process was divided into two steps: First, we conjugated the Fab@FAP with the heterobifunctional NHS-PEG-Mal; then we incubated the Fab conjugate with HFn previously labelled with FITC to be visible by flow cytometry. To optimize the nanoconstruct, we used two different types of NHS-PEG-Mal linker (5 or 10 kDa).

**Figure 3:**
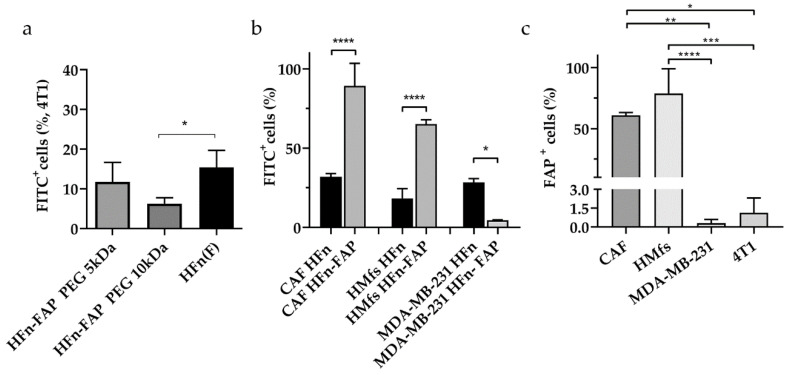
Binding with functionalized HFn-FAP nanocages. The binding of fluorescent HFn-FAP nanocages was evaluated in FAP^+^ and FAP^−^ cells and compared with the non-functionalized HFn (HFn(F)) by citofluorimetry: evaluation of the role of NHS-PEG-Mal molecular weight (5 and 10 kDa) in determining binding with FAP^−^ 4T1 cells; * *p* = 0.02 (**a**); contribution of HFn-FAP in binding FAP^+^ (CAFs and HMfs) versus FAP^−^ (MDA-MB-231) cells; * *p* = 0.0182, **** *p* < 0.0001 (**b**). FAP expression was evaluated by flow cytometry in CAFs, HMfs, MDA-MB-231, and 4T1 cells; * *p* = 0.0111, ** *p* = 0.0035, *** *p* = 0.0002, **** *p* < 0.0001 (**c**). Results are reported as average percentage of positive events ± SD of three independent experiments.

First, we evaluated whether HFn surface functionalization effectively masked HFn epitopes. This should reduce its binding with the tumor cells, in which the interaction is promoted by TfR1, the natural HFn ligand, as previously reported [36]. As it can be seen in Figure 3a, when incubating functionalized HFn-FAP 10 kDa PEG nanocages with 4T1 cells, the binding capability was significantly reduced as compared to bare HFn. This might be due to a partial masking of TfR1-binding epitopes after functionalization of the nanocage. The results obtained using 5 kDa PEG were less reproducible than the ones with 10 kDa PEG, thus suggesting more extensive masking of HFn epitopes with longer PEG. Therefore, the 10 kDa PEG was selected for the next experiments. PEG-mediated conjugation of the Fab fragment to the nanocage was confirmed by gel electrophoresis (Figure S4a). With the aim to further optimize the functionalization, we set up a reaction mix with five times more Fab fragments. The conjugation yield obtained in this case was not improved, thus suggesting that simply increasing the mole ratio in the reaction mix is not enough to increase the number of Fab moieties per single HFn (Figure S4a,b).

Then, HFn-FAP nanocages (10 kDa PEG) were incubated with FAP-overexpressing CAFs to check target recognition. Cell binding was significantly higher with functionalized HFn than with the non-functionalized protein, as shown by 89% versus 32% of CAFs that were bound by HFn-FAP and bare HFn respectively. This result suggested that the high FAP expression on these cells drives the interaction with nanoconjugated Fab (Figure 3b). 

To further prove the selective binding capability of HFn-FAP nanoparticles, we included in our analysis two additional cellular models of human origin: human-activated myofibroblasts (HMfs, Figure S3b), where we previously documented stable FAP overexpression [40], and the MDA-MB-231 breast cancer cell line (Figure S3c). Binding experiments confirmed that cells with high FAP expression (HMfs) have higher binding with functionalized HFn-FAP nanocages than with bare HFn. By contrast, cells with low levels of FAP expression (MDA-MB-231) have higher binding with bare HFn, where binding is mainly mediated by interaction with TfR1 (Figure 3b). Binding results were in line with surface expression of FAP in the cells (Figure 3c). As a confirmation, TfR1 expression was also analyzed by flow cytometry and confirmed in all cell models tested (Figure S3d). Cellular uptake of HFn-FAP was evaluated in FAP^+^ cells, showing that within 3 h of incubation, functionalized nanocages are internalized in the cell cytoplasm where they are found in close proximity to EEA1-positive endocytic vesicles (Figure S5). This observation recalls what has been already observed for HFn nanocages, which are rapidly internalized in endosomes upon receptor-mediated endocytosis [35,38].

### 3.3. Development of HNav and HNav-FAP Nanodrugs

**Figure 4:**
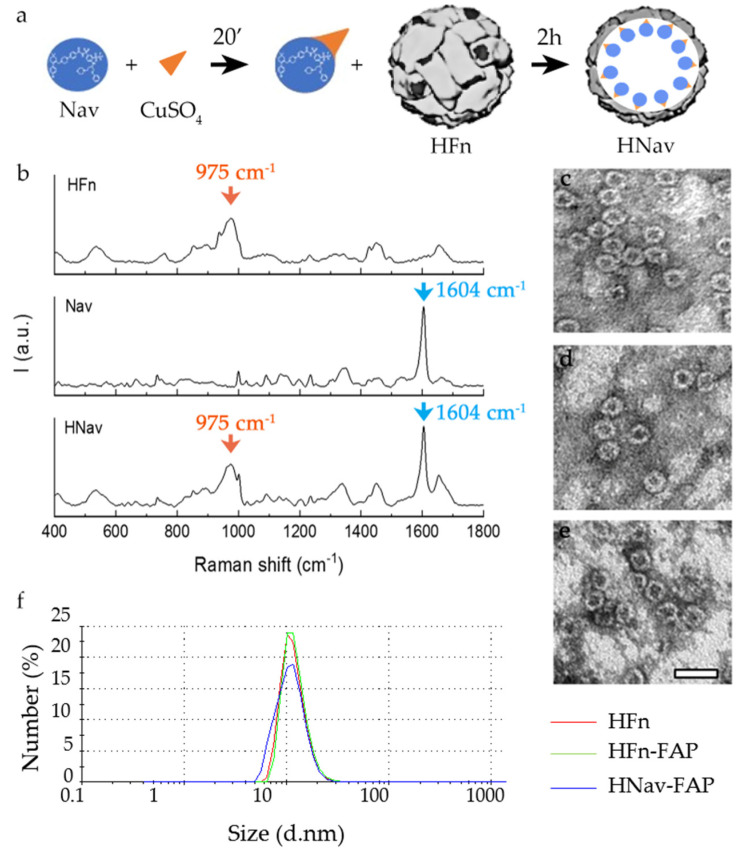
Development of HNav and HNav-FAP. Schematic representation of Nav loading into HFn: Nav (blue circle) was coupled with CuSO_4_ (orange triangle) obtaining a Cu(II)–Nav complex; the complexed drug was added to HFn (gray sphere) where it interacts thanks to the intrinsic affinity of HFn with metal ions (**a**). Raman spectra of HFn, Nav, and HNav, where the characteristic peaks of HFn (orange peak, 975 cm^−1^) and Nav (blue peak, 1604 cm^−1^) are highlighted (**b**). Transmission electron microscopy images of HFn (**c**), HNav (**d**) and HNav-FAP (**e**). Scale bar = 20 nm. Representative frequency curves of DLS analysis of HFn, HFn-FAP, and Hnav-FAP confirm that functionalization and drug loading did not modify the size properties of the nanocages (**f**).

To load Nav into HFn nanocages, we used a two-step strategy, based on the natural affinity that HFn has for metal ions. Together with the pH-based disassembly-reassembly, this is the most widely used method to prepare HFn-based nanodrugs [35]. First, Nav was coupled with anhydrous CuSO_4_ (Figure 4a). Then, the complex was incubated with HFn and, thanks to the protein affinity for metal dications, it was efficiently loaded inside the nanocage. The so-formed nanodrug (HNav) was purified from unreacted drug by gel-filtration. After purification, the functionalized nanodrug (HNav-FAP) was prepared by incubating HNav with the Fab@FAP as described above for bare HFn, avoiding any freeze-thaw process to improve stability and process yield. We quantified HFn recovery and drug encapsulation efficiency (EE%) by UPLC MS/MS after each step of the nanodrug preparation. Table 1 shows that both HFn recovery and Nav encapsulation are quite high. The highest protein loss was observed during the first step of drug encapsulation, probably due to a partial protein collapse in solution caused by high Nav hydrophobicity. In terms of Nav recovery, the encapsulation efficiency was reduced in the functionalization step. The loss during this step could be explained by a possible release of the drug during the reaction. Consequently, the average number of Nav molecules per HFn in the functionalized nanodrug was lower than in the non-functionalized one (48.7 ± 18.5 for HNav-FAP against 64.1 ± 5.7 for HNav). 

**Figure 5:**
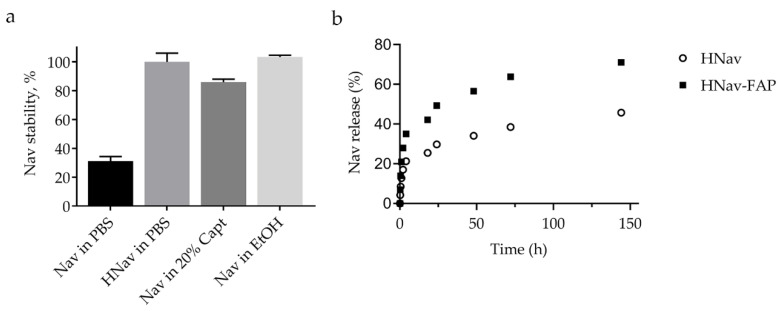
Nav and HNav solubility in water-based solvents shown as a percentage of drug recovered in solution. 0.1 M Captisol^®^ (20 %, *w/v*) was used as control carrier with solubilizing potential. An equal amount of Nav dissolved in ethanol was used as reference to set 100% solubility (**a**). Kinetics of Nav release from HNav and HNav-FAP measured by microdialysis at 37 °C, expressed as % of recovered drug (**b**).

We also characterized HNav and HNav-FAP by Raman spectroscopy, TEM and DLS. In the Raman spectra of the free drug and HFn, we found characteristic peaks of the two components at 1604 and 975 cm^−1^, respectively (blue and orange highlights, Figure 5b). The presence of both peaks in the spectra of the nanodrug confirmed the encapsulation of Nav inside the protein. Moreover, analysis of TEM micrographs of HFn, HNav, and HNav-FAP (Figure 4c–e respectively) confirmed that during the encapsulation and functionalization processes the characteristic hollow sphere nanocage structure of HFn was not modified. DLS and Z-potential (Figure 4f and Table 2) confirmed that the diameters of HFn, HFn-FAP, and HNav-FAP were all in the range of 12–13 nm, with slightly negative surface charges and that the functionalization with the FAP antibody fragments did not contribute to significant change in the characteristics of the nanocages.

To complete the characterization of the nanodrug, we assessed whether the nanoformulation improved water solubility of Nav. To do this, we measured by UPLC-MS/MS the amount of drug recovered in the supernatant of Nav and HNav preparations after synthesis (Figure 5a). Nav amount recovered in PBS solution was very low, due to the rapid precipitation of the molecule caused by its hydrophobic character. By contrast, once encapsulated into HFn nanocage, Nav stability was strongly increased, leading to almost 100% of Nav recovered in the solution. HNav stability was comparable to that obtained after adding the stabilizing agent Captisol^®^ to the Nav-PBS preparation, which was able to increase the Nav solubility up to 91%. These percentages were compared with what was obtained after dissolving Nav in EtOH, where Nav is completely soluble (100%). This result represents a promising advantage towards full preclinical assessment of Nav, considering that in vivo administration is currently hampered by the hydrophobic character of this drug.

Finally, we analyzed the kinetics of Nav release from the nanocage, performing a microdialysis experiment with both HNav and HNav-FAP. The release profiles reported in Figure 5b show that 20–25% of Nav was released within the first 2–3 h, then the release kinetics slowed down, exhibiting a hyperbolic curve profile. After 7 days at 37 °C, a certain amount of Nav was still recovered inside the microdialysis system (49% for HNav and 15% for HNav-FAP). The relatively slow release observed for both functionalized and non-functionalized nanodrugs suggested that both preparations were suitable for drug delivery purposes. 

### 3.4. The Pro-Apoptotic Activity of HNav

**Figure 6:**
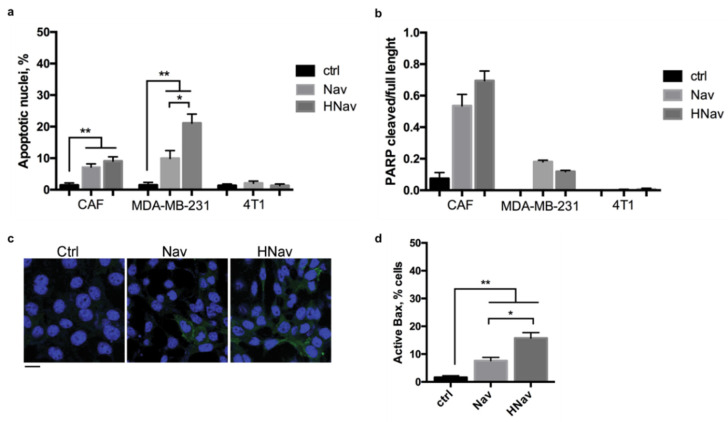
Induction of the apoptotic pathway by HNav. Percentage of apoptotic nuclei upon incubation with 1 μM Nav or HNav on CAFs, 4T1, and MDA-MB-231 cells. Nuclei were counted on at least 10 fields of view per sample; * *p* = 0.01, ** *p* < 0.005 (**a**). PARP-1 cleavage upon incubation with 1 μM Nav or HNav was calculated by densitometric analysis of Western blot bands as ratio between cleaved and full-length PARP-1 after normalization on α-tubulin (**b**). BAX activation was measured as percentage of cells stained for active BAX (green) over the total number of cells identified by DAPI staining (blue). At least 10 fields of view per sample were analyzed; * *p* = 0.02, ** *p* < 0.005. Scale bar = 10 μm (**c**,**d**). All data are shown as means ± SE (n = 3).

After the synthesis and characterization of the nanodrugs, we investigated whether Nav loading into HFn did not alter the molecular activity of the drug itself. Cells were treated with Nav or HNav, and immunofluorescence was examined to determine the pro-apoptotic effect of the nanodrug on cells in culture. Besides CAFs and 4T1 cells, we included in this analysis MDA-MB-231 as a control for human cells, which is known to be extremely sensitive to Nav, following previously documented reports [28]. As assessed by morphological analysis of cell nuclei, both Nav and HNav markedly induced apoptotic cell death in CAFs and MDA-MB-231 (Figure 6a). On the contrary, almost no effect was observed in 4T1 cells, where a very low percentage of apoptotic nuclei was found in both treatment groups. 

It has to be noted that the percentage of apoptotic nuclei in MDA-MB-231 was higher upon treatment with HNav as compared to an equal dose of free Nav. We hypothesized that this effect might be related to the specific tumor-targeting capability of HFn nanovector. By binding to the TfR1 receptor, overexpressed in many tumor cells (including the MDA-MB-231), HFn is able to rapidly deliver its payload into the target cells, working like a Trojan horse [34,38]. Through this mechanism, HNav could achieve a massive accumulation into the cell cytoplasm, where the drug exerts its activity, thus showing enhanced pro-apoptotic activity compared to the free drug. 

To confirm that treated cells were undergoing caspase-dependent apoptosis, we analyzed the caspase-dependent cleavage of the nuclear protein PARP-1, a hallmark of apoptotic cell death. Immunoblot results shown in Figure 6b and Figure S6 display the appearance of PARP-1 cleaved fraction upon treatment of CAFs and MDA-MB-231 with Nav and HNav, while control untreated cells only displayed one band corresponding to the full-length protein. 4T1 cancer cells showed no PARP-1 cleavage upon treatment with either Nav or HNav, further demonstrating no activity of the compound in these cells. 

To further investigate the molecular mechanism of action of HNav, we analyzed the activation of the pro-apoptotic protein BAX in CAFs treated with Nav or HNav. The immunofluorescence revealed that both Nav and HNav induced BAX activation in treated cells, thus confirming activation of the mitochondrial pathway (Figure 6c,d). A higher percentage of cells stained for active BAX was observed upon HNav compared to Nav alone, likely suggesting different kinetics of activity for HNav that may deserve further investigations in the future.

### 3.5. HNav-FAP Enhances Therapeutic Potential of Nav against FAP-Overexpressing Fibroblasts

**Figure 7:**
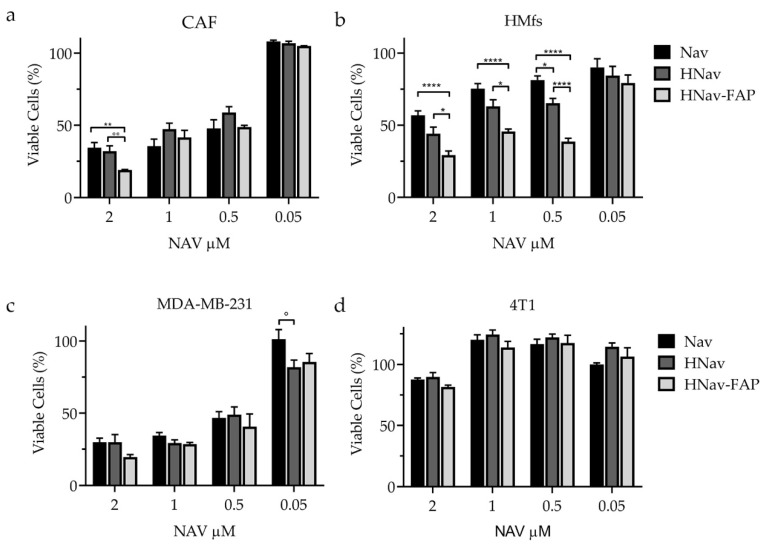
Cell viability upon treatment with HNav-FAP. CAFs (**a**), HMfs (**b**), MDA-MB-231 (**c**), and 4T1 (**d**) cells were treated with increasing concentrations of Nav, HNav, or HNav-FAP for 24 h. Viability data are reported as average percentage ± SE after normalization on untreated cells (n ≥ 3). ° *p* = 0.014, °° *p* = 0.0048, * *p* = 0.02, ** *p* = 0.0015, **** *p* < 0.0001.

To study whether the elevated binding with HFn-FAP observed in FAP^+^ cells could be translated into a higher drug efficacy, we evaluated cell viability after 24 h of incubation with Nav, HNav and HNav-FAP. Data reported in Figure 7a,b confirmed that HNav-FAP was significantly more active than HNav and the free drug in FAP-overexpressing cell models (CAFs and HMfs). In CAFs, significant differences were observed for HNav-FAP at 2 µM, with only 19% of viable cells, as compared to 32% and 35% when incubated with HNav and Nav, respectively. Similarly, HNav-FAP significantly inhibited cell viability more than HNav and Nav in HMfs. Here, the advantage of HNav-FAP was observed even at lower concentrations, down to 0.5 µM, probably due to higher FAP expression in these cells. As a control, we incubated HMfs with equivalent concentrations of bare HFn and HFn-FAP, and we found that the empty nanocages did not induce any toxic effect in the cells (Figure S7). These results also confirmed that targeting FAP by HFn-FAP does not have any effect on cell viability. 

No differences in cell viability were observed in FAP^−^ 4T1 and MDA-MB-231 cells, where the three formulations had similar activity profiles (Figure 7c,d). Comparing the different cells, MDA-MB-231 cells were the most sensitive to Nav activity with no differences among the three formulations down to 0.5 µM drug concentration; only at the lowest 0.05 µM concentration, HNav was significantly more active than free Nav (Figure 7c). The viability of resistant 4T1 was only slightly affected at the highest concentration of 2 µM for all the different preparations (Figure 7d). 

To exclude any influence in cell viability caused by CuSO_4_ used to prepare the nanodrugs, we incubated the highly Nav-responding MDA-MB-231 with equivalent concentrations of CuSO_4_ (either bare or in combination with HFn), without finding any effect on cell viability and metabolism (Figure S8).

These results confirmed the reliability of the FAP-targeting strategy in enhancing selective drug efficacy in cells expressing high levels of the target protein, while the functionalization did not improve the nanodrug efficacy in FAP^−^ cells.

**Figure 8:**
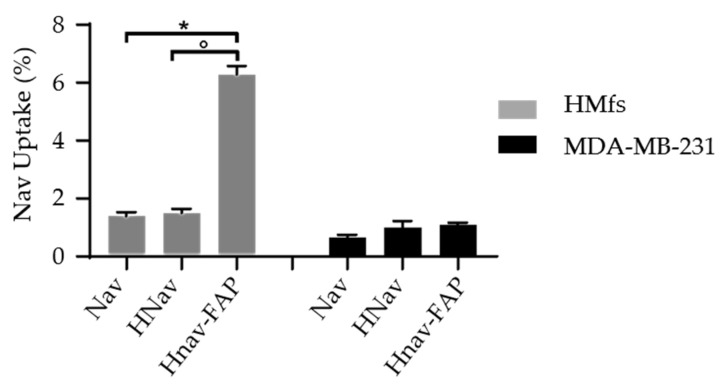
Cellular uptake of Nav. HMfs (gray bars) and MDA-MB-231 (black bars) cells were incubated with free Nav, HNav, or HNav-FAP for 1 h. Intracellular Nav was quantified by UPLC MS/MS and expressed as the intracellular percentage of the incubated dose. * *p* = 0.0314, ° *p* = 0.0344. Data are reported as average ± SD (n = 3).

Next, we studied if the different viability observed upon treatment with HNav-FAP was correlated with a higher drug uptake in FAP-overexpressing cells. HMfs and MDA-MB-231 cells were incubated with 1 µM Nav, HNav, or HNav-FAP, and the intracellular drug concentration was measured by UPLC/MS-MS analysis after 1 h. Figure 8 shows that after 1 h incubation, HNav-FAP intracellular concentration was four-fold higher than Nav and HNav in FAP^+^ HMfs cells. This initial “burst” could be due to the active role of anti-FAP functionalization that allows a more rapid uptake of the drug inside target cells. In contrast, the intracellular Nav concentration in FAP-negative MDA-MB-231 cells measured after treatment with HNav-FAP was similar to the one observed with Nav and HNav. This result suggests that FAP targeting plays an active role in the cellular uptake of the nanodrug only in FAP^+^ cells. 

### 3.6. HFn-FAP Biodistribution in a Syngeneic Model of TNBC

**Figure 9:**
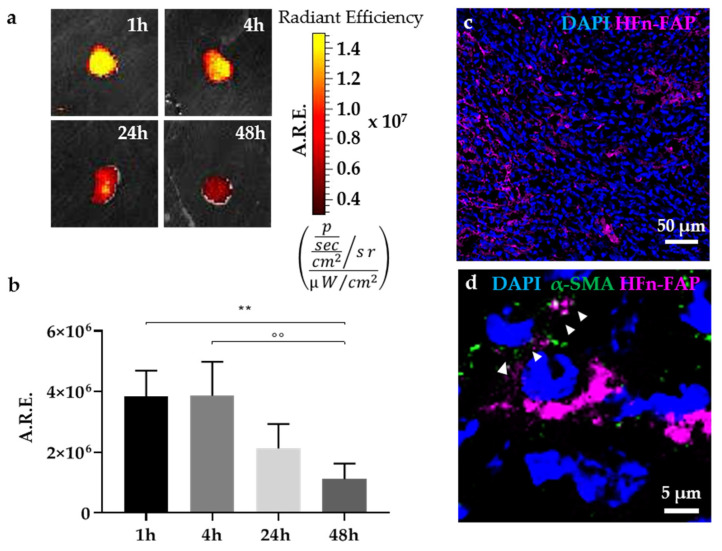
Biodistribution and tumor targeting of HFn-FAP in a TNBC model. (**a**) Ex vivo imaging of 4T1 excised tumors 1, 4, 24, and 48 h after IV administration of fluorescently labelled HFn-FAP (5mg/kg). Average Radiant efficiency (A.R.E.), color scale min = 3 × 10^6^, ma× = 1.5 × 10^7^. (**b**) Quantification of the fluorescent signal measured in the tumors as average radiant efficiency (average ± SD, n = 4); ** *p* = 0.0088, °° *p* = 0.0048. (**c**) Confocal Microscopy image of a tumor section 1 h after HFn-FAP administration confirm intratumoral distribution of the nanocages (purple signal); cell nuclei were stained with DAPI (blue). (**d**) Immunofluorescence analysis of α-SMA (green) suggest that HFn-FAP (purple) are able to target CAFs; cell nuclei were stained with DAPI (blue); Scale bars = 20 µm (panel **c**) and 5 µm (panel **d**).

To preliminarily evaluate the potential of our nanoconstruct in vivo, we injected fluorescently labelled HFn-FAP into the tail vein of 4T1 tumor-bearing mice and monitored their biodistribution by fluorescence imaging at 1, 4, 24, and 48 h. The tumor targeting of HFn-FAP was clearly appreciated by ex vivo imaging of tumors (Figure 9). We observed a strong intratumoral signal at 1 h and 4 h after administration, while the fluorescence progressively decreased in intensity at 24 h and 48 h post-injection (Figure 9a,b). The intratumor distribution of HFn-FAP was confirmed by confocal microscopy, where a specific signal throughout the whole section was observed (in purple, Figure 9c). To get a preliminary evaluation of whether the functionalization allowed HFn-FAP to target CAFs in vivo, we stained tumor sections with the fibroblast marker α-SMA. A partial co-localization of the nanocage signal (purple) with α-SMA signal (in green) was observed in some cells, thus supporting the targeting capability of HFn-FAP (white arrowheads, Figure 9d).

Systemic biodistribution of HFn-FAP demonstrated that nanoparticles were also captured by the liver during the first hours post-injection, as shown by intense fluorescence signal in this organ that almost covered the fluorescence of the tumor when imaging the mice in vivo (Figure S9). HFn-FAP were rapidly metabolized by the liver after 4 h, with merely detectable signal in the liver 24 h later (Figures S9 and S10). An intense fluorescent signal was also detected in the bladder of mice and in the urine samples at 1 h and 4 h, thus indicating a clearance by the urinary tract (Figure S9). Accordingly, some detectable signal was recorded in the kidneys excised at 1 h post-injection (Figure S10). 

We compared our results with the ones published for non-functionalized HFn [41] and found very similar biodistribution and clearance kinetics, suggesting that the functionalization with Fab@FAP moiety does not modify the overall biodistribution and clearance of the particles.

## 4. Discussion

Many types of nanoparticulate delivery systems have been introduced preclinically for the development of smart anti-cancer treatments. The main advantages of this approach are (i) promoting specific target delivery by surface functionalization of the nanoagents, thus reducing side effects, and (ii) allowing the encapsulation of insoluble drugs, thus making them usable for parenteral administration [42,43]. In this study, we combined both advantages and developed new HFn-based nanodrugs functionalized with FAP-targeting ligands able to enhance Navitoclax delivery into FAP-overexpressing CAFs target cells. 

In fact, several works have highlighted the key role that CAFs play in promoting cancer progression, invasiveness and metastasis in many types of solid tumors, including breast cancer [44,45]. For these reasons, the development of safe and reliable strategies able to target CAFs could be a powerful approach to help control growth and spreading of many types of aggressive cancers [46,47]. Pro-tumorigenic CAFs display overexpression of the membrane protein FAP. This is why FAP is emerging as a promising antigen for smart CAF-targeted therapeutic strategies [48,49]. Different immunotherapeutic agents have been developed to block FAP proteolytic activity, thus preventing tumor growth and proliferation. However, therapeutic efficacy has not met initial expectations and clinical translation is still very limited, probably due to the small impact of such strategy in the overall complexity of phenomena regulating tumor growth [17,18,50]. More promising results are emerging using FAP as a target moiety to prompt selective delivery of cytotoxic agents into CAFs. Few groups have developed nanoparticulate delivery systems functionalized with anti-FAP antibodies (either entire or fragments) to promote CAF specific delivery of cytotoxic agents, molecular inhibitors, or contrast agents [10]. 

In this study, we coupled the FAP-targeting specificity with the selective activity of Nav, an experimental drug with increased efficacy in CAFs and myofibroblast rather than in cancer cells. In particular, in TNBC, Nav shows very limited efficacy due to innate drug resistance [51]. Our results show that by functionalizing HFn with FAP antibody fragments, we were able to significantly increase CAF tropism of the nanocages and, at the same time, reduce their off-target distribution to tumor cells, most likely by competing with the natural TfR1 binding site exploited by HFn to interact with cells. This result is very promising for developing new targeted therapies. 

We decided to investigate the targeting and cytotoxic properties of engineered nanodrugs in cells of both mouse and human origin for different reasons. The first one is gaining important information for the set-up of a reliable preclinical model, in which we are going to test the efficacy of our nanodrugs. Thanks to the strong targeting observed in CAFs and the concomitant reduced uptake in 4T1 cells, we are confident that our anti-FAP functionalized nanodrugs could be reliable agents for delivering cytotoxic drugs specifically into CAFs also in vivo. The second reason is giving a stronger translational direction to our results. Our data showing that HFn-FAP is able to selectively target human activated myofibroblasts further supports the idea that our nanodrug could be applied not only to CAFs in TNBC, but also to other malignancies where stromal FAP is overexpressed. Finally, our results confirm that the higher efficacy of functionalized nanocages in FAP^+^ cells was correlated with a higher intracellular uptake of such particles. 

Our in vivo data suggest that the biodistribution profile observed for HFn-FAP was similar to what we previously observed with non-functionalized HFn. Here, the intratumoral signal remained highly stable up to 4 h upon administration, while the elimination profile from off target organs was much more rapid as compared to the one in the tumor. This, together with the immunofluorescence studies proving HFn-FAP targeting of CAFs in vivo, suggest that the functionalization with FAP is able to trigger a specific CAF recognition in the tumor that, at the same time, might also enhance intratumoral retention of the particles.

Results from our study also document effective nanoformulation of Nav drug. Encapsulated Nav is released inside the cells and maintains its native pro-apoptotic activity in sensitive cells, as verified by apoptosis induction, BAX activation, and PARP-1 cleavage. This was not taken for granted, as after coupling with copper sulfate, the properties of the drug might have changed. Considering the intrinsic difficulties in loading such hydrophobic drugs, this method allowed us to load enough drug to have a strong specific activity on target cells, without disassembling the structure of HFn and ensuring more robust stability to the drug. Further studies are needed to validate our hypothesis that HNav-FAP can be employed to eradicate CAFs in combination with tumor cells-targeted therapies. Finally, it will be interesting to verify if Nav encapsulation effectively reduces its strong known side effects, in particular thrombocytopenia. We are confident that, thanks to the high intrinsic biocompatibility and favorable pharmacokinetics of HFn, and most importantly to tumor tropism of HFn nanocages both in vitro and in vivo, Nav release in the blood will be unlikely, thus reducing contact time with platelets and related side effects. 

In conclusion, our results give us confidence that the use of HNav-FAP in combination with a chemotherapeutic, would allow us to develop a double strategy with selective efficacy on CAFs and tumor cells, thus maximizing therapeutic potential and reducing side effects. Moreover, as the 4T1 preclinical model normally leads to the formation of metastases, it would be extremely interesting to study whether the CAF-targeting nano-strategy could reduce the formation of metastases.

## Figures and Tables

**Table 1:** Yields of HFn recovery and encapsulation efficiency after Nav loading.

	HFn Recovery, %	EE, %	Nav/HFn
HNav ^1^	61.8 ± 3.8	72.1 ± 5	64.1 ± 5.7
HNav-FAP ^2^	68.1 ± 17.2	54.7 ± 18.7	48.7 ± 18.5

^1^ n = 27, ^2^ n= 9.

**Table 2:** Size and surface charge of nanocages evaluated by DLS and Z-pot.

	Size (d.nm)	Z-pot (mV)
HFn	13.02 ± 3.1	−11.6 ± 5.6
HFn FAP	11.69 ± 3.2	−12.6 ± 6.9
Hnav-FAP	13.42 ± 3.0	−10.2 ± 4.4

## Data Availability

Data are available in a publicly accessible repository https://doi.org/10.13130/RD_UNIMI/SKRLH0 (accessed on 2 February 2021) after publication.

## Supplementary Materials

The following are available online at https://www.mdpi.com/2073-4409/10/2/328/s1, Figure S1: representative images of healthy and apoptotic nuclei upon Nav and HNav treatment, Figure S2: flow cytometry analysis of CAFs isolation from 4T1 tumor, Figure S3: characterization of the cellular models used in the study, Figure S4: Proof of Fab@FAP conjugation to HFn nanocage, Figure S5: Confocal microscopy of HFn-FAP internalized in HMfs, Figure S6: immunoblotting of PARP-1 cleavage upon Nav and HNav treatment, Figure S7: Cytotoxicity of functionalized HFn-FAP and non-functionalized HFn nanocages, Figure S8: Cytotoxicity of CuSO_4_ and HFn in MDA.MB.231 cells, Figure S9: Biodistribution of HFn-FAP in a murine model of TNBC, Figure S10: Ex vivo imaging of off target organs upon injection of HFn-FAP.
