# Supplementary material for: Selective Targeting of Cancer-Associated Fibroblasts by Engineered H-Ferritin Nanocages Loaded with Navitoclax
Source: Cells. 2021 Feb 5;10(2):328. doi: 10.3390/cells10020328 (PMC7915356; doi:10.3390/cells10020328)
Supplement: Supplementary file 1 [file cells-10-00328-s001.pdf]

cells

Article

Selective targeting of Cancer-Associated Fibroblasts by engineered H-ferritin nanocages loaded with navitoclax

Supplementary Materials

Leopoldo Sitia [1], Arianna Bonizzi [1], Serena Mazzucchelli [1], Sara Negri [2], Cristina Sottani [2], Elena Grignani [2], Maria Antonietta Rizzuto [3], Davide Prosperi [3], Luca Sorrentino [4], Carlo Morasso [2], Raffaele Allevi [1], Marta Sevieri [1], Filippo Silva [1], Marta Truffi [2,*] and Fabio Corsi [1,2,*]

[1] Department of Biomedical and Clinical Sciences "L. Sacco", University of Milan, 20157 Milan, Italy; leopoldo.sitia@unimi.it, arianna.bonizzi@unimi.it, serena.mazzucchelli@unimi.it, raffaele.allevi@unimi.it, marta.sevieri@unimi.it, filippo.silva@unimi.it
[2] Istituti Clinici Scientifici Maugeri IRCCS, 27100 Pavia, Italy; sara.negri@icsmaugeri.it, cristina.sottani@icsmaugeri.it, elena.grignani@icsmaugeri.it, carlo.morasso@icsmaugeri.it, marta.truffi@icsmaugeri.it
[3] Department of Biotechnology and Biosciences, University of Milan-Bicocca, 20126 Milan, Italy; m.rizzuto3@campus.unimib.it, davide.prosperi@unimib.it
[4] Colorectal Surgery Unit, Fondazione IRCCS Istituto Nazionale dei Tumori di Milano, 20133 Milan, Italy; luca.sorrentino@istitutotumori.mi.it
* Correspondence: fabio.corsi@unimi.it, fabio.corsi@icsmaugeri.it, Tel.: +39 0250319850-19858 (F.C.); marta.truffi@icsmaugeri.it, Tel: +39 0382592219 (M.T.)

Citation: Sitia, L.; Bonizzi, A.; Mazzucchelli, S.; Negri, S.; Sottani, C.; Grignani, E.; Rizzuto, M.A.; Prosperi, D.; Sorrentino, L.; Morasso, C.; et al. Selective Targeting of Cancer-Associated Fibroblasts by Engineered H-Ferritin Nanocages Loaded with Navitoclax. *Cells* **2021**, *10*, 328. https://doi.org/10.3390/cells10020328

Academic Editor: Sergio Comincini
Received: 15 November 2020
Accepted: 2 February 2021
Published: 5 February 2021

Publisher's Note: MDPI stays neutral with regard to jurisdictional claims in published maps and institutional affiliations.

Copyright: © 2021 by the authors. Licensee MDPI, Basel, Switzerland. This article is an open access article distributed under the terms and conditions of the Creative Commons Attribution (CC BY) license (http://creativecommons.org/licenses/by/4.0/).

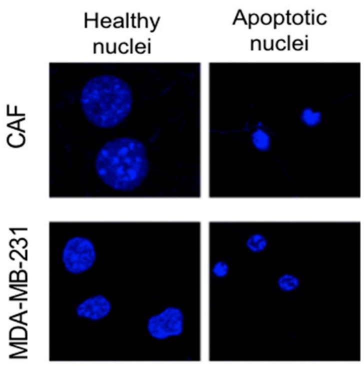

Figure S1. Micrographs of healthy and apoptotic nuclei upon treatment of CAFs and MDA-MB-231 cells.

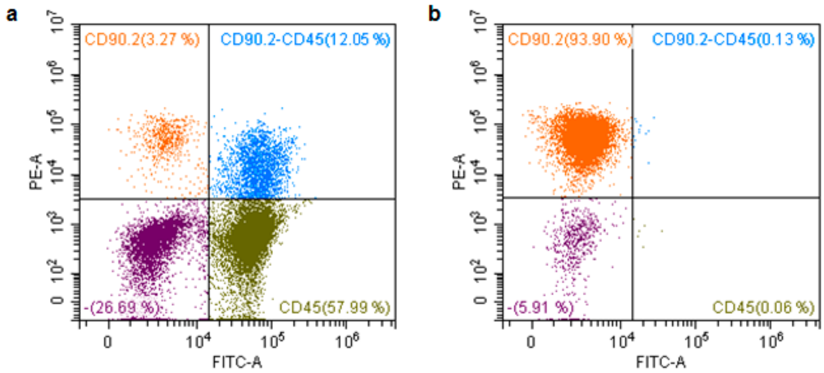

Figure S2: Flow cytometry analysis of the first two steps performed to isolate CAFs from 4T1 dissociated tumors: Tumor dissociation and removal of red blood cells (**a**). Pre-enrichment by non-tumor-associated fibroblast depletion cocktail (**b**). Staining was performed with anti-CD45-FITC and anti-CD90.2-PE antibodies

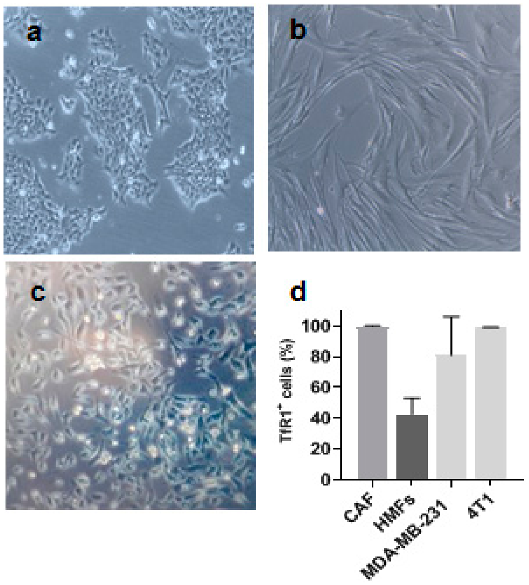

Figure S3: Morphology of cellular models used in the study: 4T1 (**a**), HMfs (**b**) and MDA-MB-231 (**c**) cells by brightfield microscopy; expression of TfR1 analyzed on CAFs, 4T1, HMfs and MDA-MB-231 cells by flow cytometry (**d**).

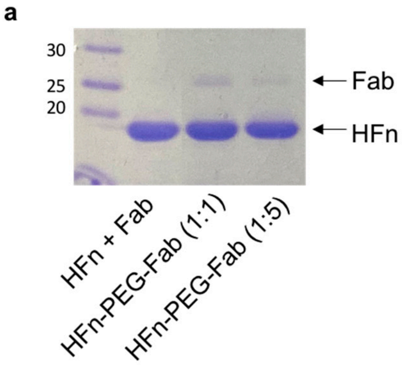

b

Samples	Fab/HFn RATIO
HFn + Fab 1:1	0,013
HFn-PEG-Fab 1:1	0,370
HFn-PEG-Fab 1:5	0,301

Figure S4: Proof of Fab@FAP conjugation to HFn nanocage: conjugated (HFn-PEG-Fab, PEG 10 kDa molecules were pre-conjugated to the Fab@FAP fragment) and surface adsorbed (HFn+Fab, without previous conjugation of Fab@FAP with PEG) samples were diluted in sample buffer containing 2% SDS and 4% β-mercaptoethanol and boiled 10 min before loading in the gel. Samples were separated by SDS-PAGE in a 12% running gel and a 4% stacking gel. PageRuler Unstained protein ladder (Thermo Fisher Scientific) was used as protein marker (first lane). After the electrophoretic separation, the gel was stained with Imperial Protein Stain (Thermo Fisher Scientific) to display Fab@FAP and HFn bands (**a**). Densitometric analysis of the bands revealed that PEG conjugation (HFn-PEG-Fab 1:1 and 1:5) increases the amount of Fab@FAP per HFn as compared to the unspecific binding (HFn+Fab 1:1). There is no

advantage in increasing the HFn:Fab ratio, as the amount of conjugated Fab@FAP per HFn does not increase in case of HFn-PEG-Fab 1:5 versus HFn-PEG-Fab 1:5. The densitometric analysis of protein bands was performed using Fiji software (**b**).

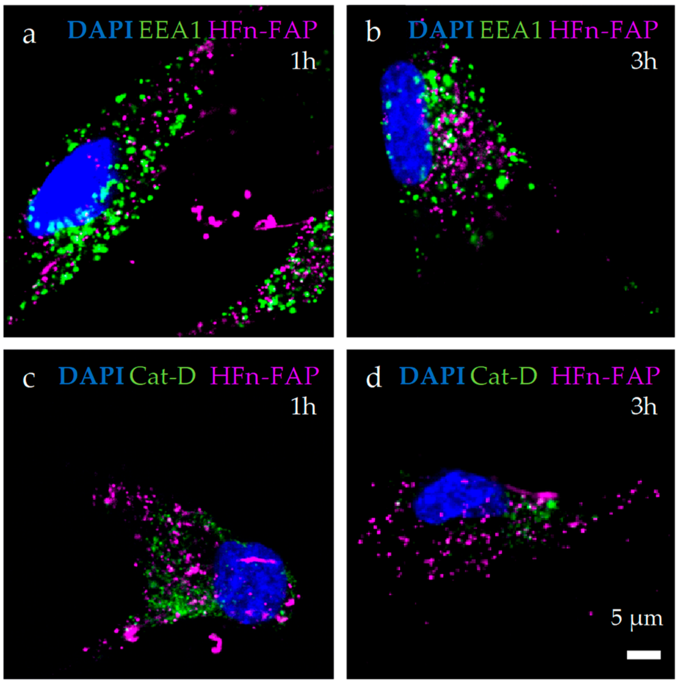

Figure S5: Confocal microscopy of HFn-FAP (purple) intracellular signal in HMfs. Partial colocalization with early endosomes (EEA1 green, **a** and **b**) was found after 1 and 3 hours of incubation. HFn-FAP colocalization with lysosomes (Cat-D, green **c** and **d**) was less evident.

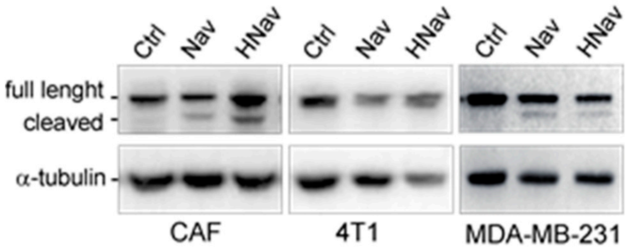

Figure S6: Immunoblot of PARP-1 proteolytic cleavage upon Nav or HNav treatment on CAFs, 4T1 and MDA-MB-231. Whenever active, the drug induces the formation of a PARP-1 fragment of 89 kDa appearing below the full-length protein band.

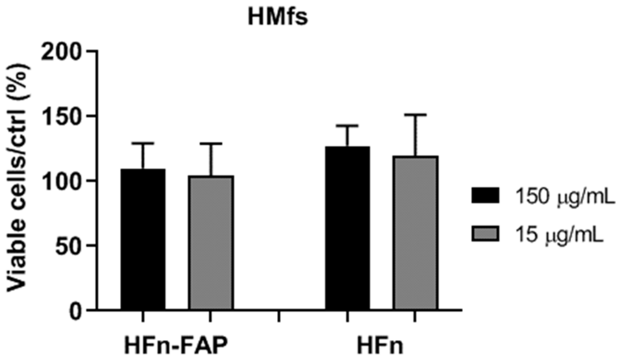

Figure S7: Cytotoxicity of functionalized HFn-FAP and non-functionalized HFn nanocages, in HMfs (FAP+) cells. Results, reported as % of viable cells in comparison to untreated control cells, showed no significant effect on cell viability at all tested concentrations. Concentrations used here were selected according to those ones used for pharmacological studies with Nav loaded nanodrugs (10 and 1 µM of Nav respectively). These results confirm that the effect observed with HNav and HNav-FAP on these cells at a drug concentration of 1 µM are only due to a Nav- related activity, while FAP only has a targeting function. Results are reported as average ± SD of 6 independent measurements.

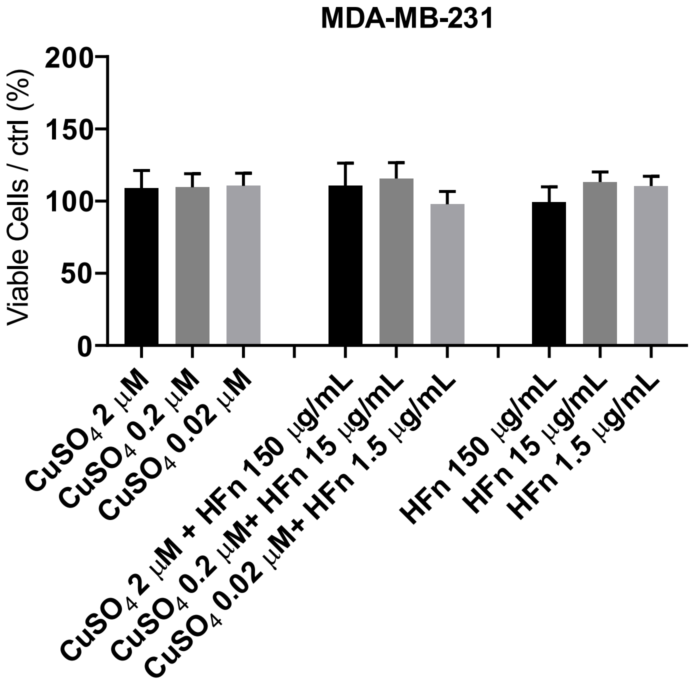

Figure S8: Cytotoxicity of CuSO4 and HFn in MDA.MB.231 cells. These results confirm that neither the copper sulfate used for drug complexation, nor to the empty HFn nanocarrier, nor the combination of the two, have any significant cytotoxic effect. Concentrations used here were selected according to the ones used for pharmacological studies with Nav loaded nanodrugs and correspond to 10, 1 and 0.1 µM of Nav. Results are reported as average ± SD of 6 independent measurements.

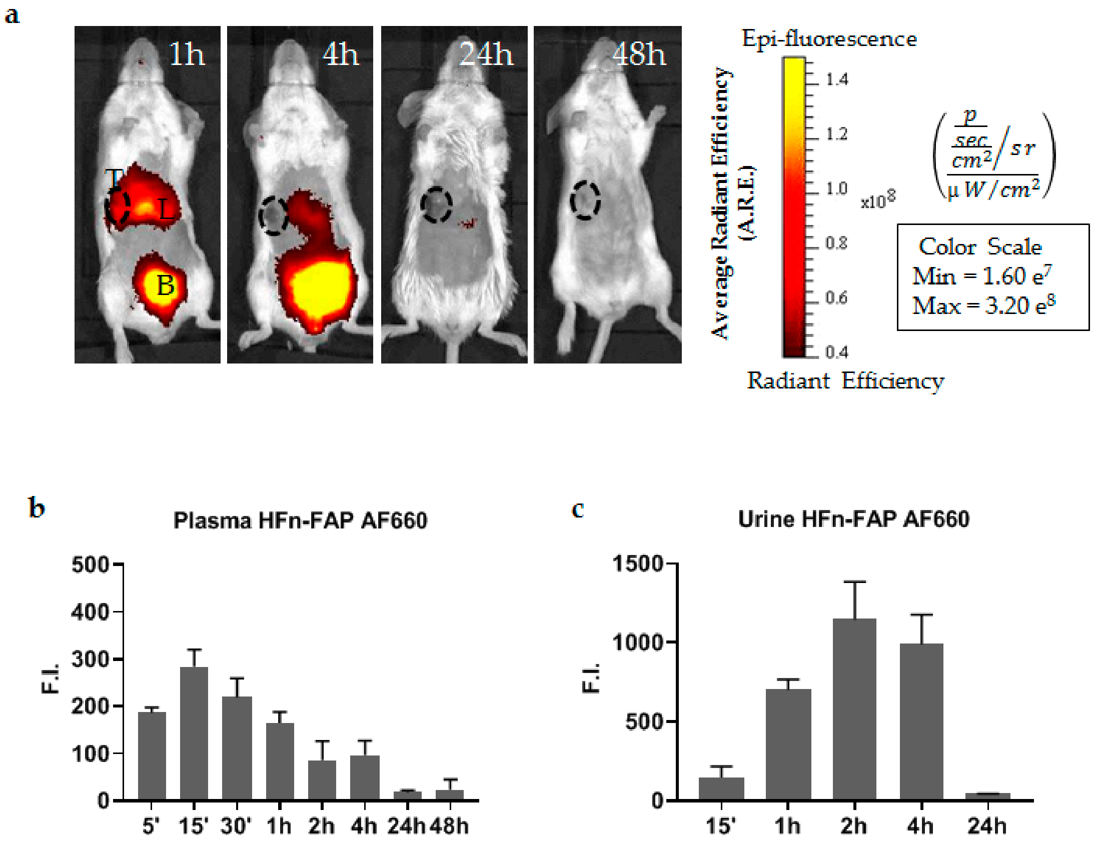

Figure S9: Biodistribution of fluorescently labelled HFn-FAP. In vivo imaging analysis performed 1h, 4h, 24h and 48h after IV administration showed a strong signal in the liver and the bladder of treated mice, up to 4h after administration. The signal was merely visible in the liver 24h later and dropped below limit of detection in all other organs (**a**). Fluorescence analysis of plasma revealed a peak of HFn-FAP signal 15′ after administration, with a detectable signal until the 4-hour time point. The signal measured at 24h and 48h was close to the baseline and in some cases below the limit of detection (**b**). The fluorescence in urine, confirm HFn-FAP elimination through the urinary tract within the first 4 hours of analysis (**c**).

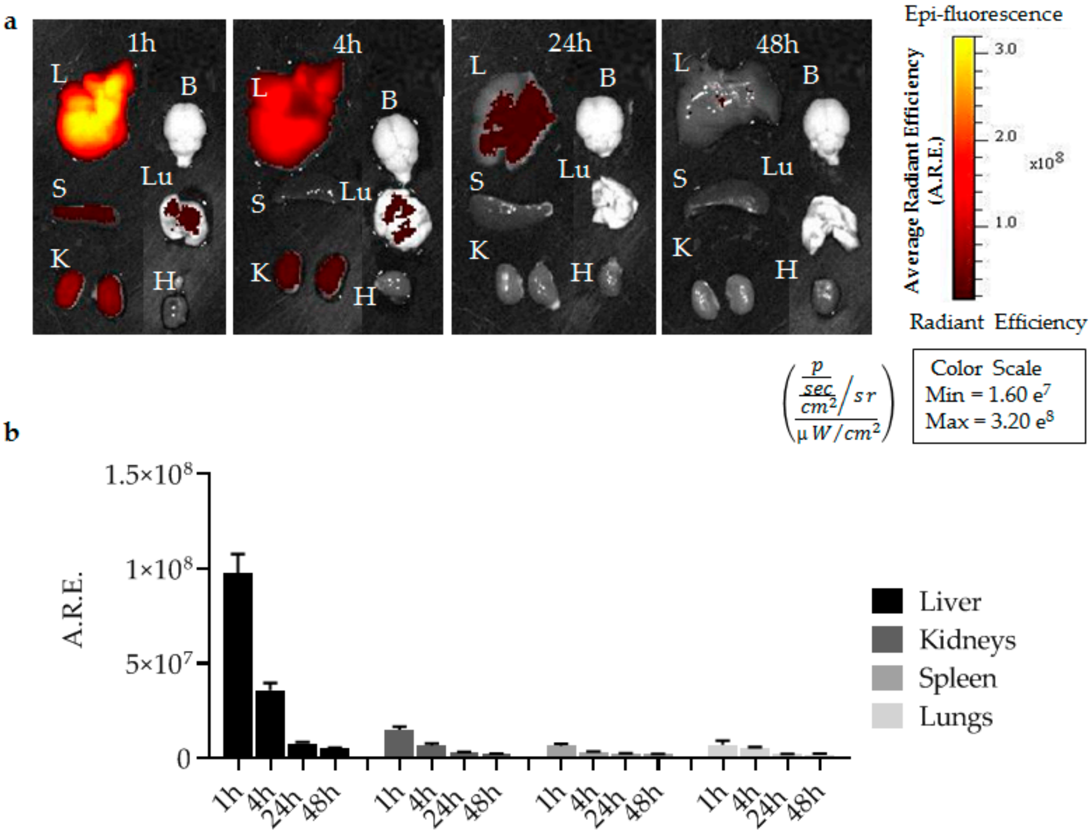

Figure S10: Biodistribution in off target tissues. *Ex vivo* imaging of liver (L), kidneys (K), spleen (S), Lungs (Lu), brain (B) and heart (H) of treated mice, 1h, 4h, 24h and 48h after HFn-FAP administration confirmed the major findings obtained *in vivo*, with the strongest fluorescence found in liver followed by a milder signal in kidneys, involved in HFn-FAP renal filtration, spleen and lungs (**a**). The fluorescence signal in liver, kidneys, spleen and lungs was quantified and reported as average radiant efficiency (A.R.E.) (**b**). Brain and heart were not included in the histogram, as their fluorescence remained below the detection limit at all time points.
